# Recognising and diagnosing Cushing’s syndrome in primary care: challenging but not impossible

**DOI:** 10.3399/bjgp22X720449

**Published:** 2022-07-29

**Authors:** Kate Scoffings, Damian Morris, Andrew Pullen, Sharon Temple, Anna Trigell, Mark Gurnell

**Affiliations:** Huntingdon Road Surgery, Cambridge.; East Suffolk & North Essex Foundation Trust, Department of Diabetes & Endocrinology, the Ipswich Hospital, Suffolk.; Lavant Road Surgery, Chichester.; Spinney Surgery, St Ives.; Buckden Dermatology Clinic Community Services, Buckden.; Wellcome — MRC Institute of Metabolic Science, University of Cambridge, and NIHR Cambridge Biomedical Research Centre, Addenbrooke’s Hospital, Cambridge.

## BACKGROUND

The catalyst for this article was the experience of one of the authors whose diagnosis of Cushing’s was not immediately recognised despite the presence of numerous suggestive clinical features and comorbidities. This prompted the authors to convene a group of primary and secondary care clinicians to explore how to increase awareness and simplify the process of recognising and diagnosing Cushing’s syndrome (CS) in primary care.

## WHY IS IT IMPORTANT NOT TO OVERLOOK CUSHING’S SYNDROME?

Endogenous CS has traditionally been considered a rare condition, with an estimated annual incidence of 1.8 to 3.2 cases per million population,^1^ although a recent study suggests the true incidence may be considerably higher, especially in individuals aged <65 years.^2^ CS can be classified as either adrenocorticotrophic hormone (ACTH)-dependent or ACTH-independent. ACTH-dependent CS is caused by a pituitary adenoma (also known as Cushing’s disease) or an ectopic ACTH-producing tumour. In the absence of exogenous corticosteroid therapy, ACTH-independent CS points to an adrenal origin. CS is an important diagnosis not to overlook because of its significant physical and psychological morbidities, associated comorbidities, and the excess mortality that complicates uncontrolled hypercortisolism (prior to effective treatments becoming available, 5-year mortality rate was 50% after symptom onset).^3^

## WHEN SHOULD THE POSSIBILITY OF ENDOGENOUS CUSHING’S SYNDROME BE CONSIDERED IN PRIMARY CARE?

CS can present at any age, but specific subtypes are more common in certain patient groups, for example, a younger woman is more likely to have a pituitary origin, whereas the possibility of ectopic disease is increased in an older smoker.

**Figure 1.**
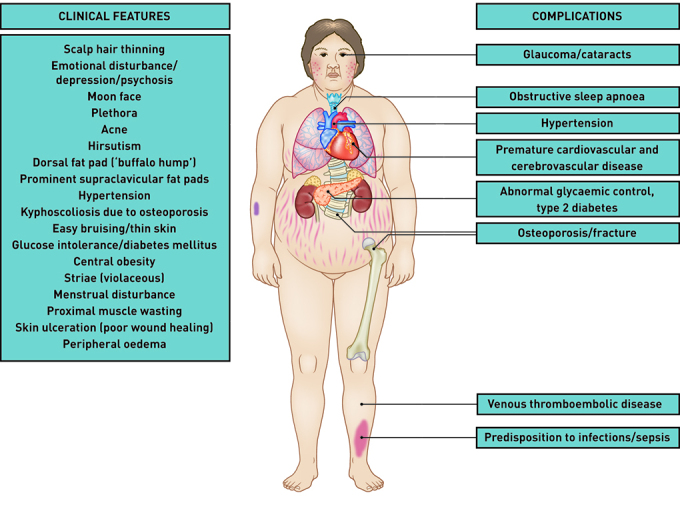
*Cushing’s syndrome: clinical features and associated comorbidities.*

The clinical manifestations of the classical Cushingoid phenotype are shown in Figure 1, together with associated complications. While this figure portrays a florid example of a patient with CS, in practice many patients will present with a more subtle constellation of features, which may only be fully appreciated once the underlying hypercortisolism has been corrected (Supplementary Figure 1). Alternatively, reviewing old photographs on a patient’s phone may provide clear evidence that their appearance has changed over time.

An important factor in the delayed diagnosis reported by many patients is the failure of healthcare professionals to recognise the significance of various combinations of symptoms or signs and linked comorbidities. For example, early-onset hypertension, diabetes mellitus, venous thromboembolism (VTE), and/or osteoporosis in a young woman should prompt consideration of CS.^4^^,^^5^ Similarly, a number of discriminatory dermatological (for example, striae [especially violaceous], spontaneous bruising, thin skin, new-onset acne in adults, with facial plethora and/or moon face) and musculoskeletal (for example, muscle wasting with proximal myopathy) manifestations should also trigger the clinician to explore the possibility of hypercortisolism.^5^

## WHICH INVESTIGATIONS ARE MOST APPROPRIATE TO SCREEN FOR CUSHING’S SYNDROME IN GENERAL PRACTICE?

**Figure 2.**
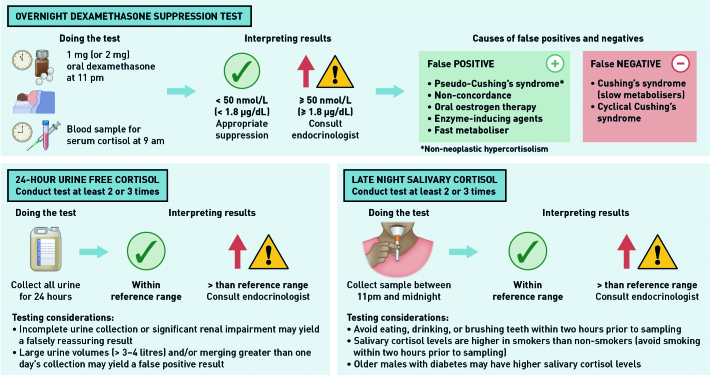
*Screening tests for Cushing’s syndrome.*

There is no single perfect test for CS. Accordingly, guidelines often offer a menu of options, each with potential advantages and disadvantages; most commonly these are the overnight dexamethasone suppression test (O/N DST), 24-hour urine free cortisol (UFC) estimation, or late-night salivary cortisol (LNSC) measurement.^6^ Figure 2 describes how to perform and interpret these tests, and outlines some important considerations when choosing which to deploy. Importantly, although the O/N DST is sometimes viewed as challenging to conduct, it is a safe and effective investigation for use in the primary care setting and is the preferred screening investigation for many endocrinologists. The O/N DST should be distinguished from the two 48-hour dexamethasone suppression tests: 1) low-dose DST (LDDST), which may also be used to confirm or exclude a diagnosis of CS, and 2) high-dose DST (HDDST), which is used to distinguish pituitary-driven from ectopic CS.

## WHEN SHOULD A PATIENT BE REFERRED TO SECONDARY CARE?

CS may be difficult to diagnose at first presentation because of the non-specific nature of some features, and frequently the diagnosis becomes more apparent as the condition evolves; as such, a period of observation may be necessary/appropriate. When deciding whether to refer to an endocrinologist, it can be helpful to consider the probability of endogenous CS based on clinical index of suspicion (low, intermediate, or high), together with the results of an initial screening investigation, for example:

- low probability plus negative screening test = unlikely to require referral;
- low probability but positive screening test (with no explanation for false positive) = refer; and
- intermediate to high probability = arrange screening test and refer.

## WHAT NEW DEVELOPMENTS ARE ON THE HORIZON TO ENHANCE THE DIAGNOSIS OF CUSHING’S SYNDROME IN PRIMARY CARE?

Rare diseases are not always front of mind for GPs and other healthcare professionals working in primary care, and it is not practical to screen every patient who presents with these common symptoms. As such, automated digital systems could play a role in identifying potential cases of CS that may otherwise be overlooked. Future tools in this regard could include the integration of sensitive algorithms into patient record systems such as EMIS (EMIS Group, Leeds, UK) and SystmOne (TPP, Leeds, UK), providing red flags when a certain pattern of clinical features is recorded. Others have advocated the use of computer software to analyse photographs collected over time to identify morphological changes of the head and face that may suggest CS, although it could be argued that in this setting the key step of considering the possibility of CS has already occurred.^7^

## KEY TAKEAWAY MESSAGES

A potential diagnosis of CS should not be overlooked given the ramifications of uncontrolled hypercortisolism. Although investigation for possible CS is frequently perceived as challenging, the initial steps are more straightforward than often anticipated, and entirely feasible to conduct in the primary care setting.
